# BARRIERS TO DISEASE SELF-CARE AMONG OLDER NON-HISPANIC BLACK AND HISPANIC MEN WITH CHRONIC CONDITIONS

**DOI:** 10.1093/geroni/igad104.2531

**Published:** 2023-12-21

**Authors:** Matthew Smith, Caroline Bergeron, Ledric Sherman, Oluyomi Oloruntoba, Ashley Merianos

**Affiliations:** Texas A&M University, College Station, Texas, United States; University of Ottawa, Ottawa, Ontario, Canada; Texas A&M University School of Public Health, College Station, Texas, United States; Texas A&M University, College Station, Texas, United States; University of Cincinnati, Cincinnati, Ohio, United States

## Abstract

Disease self-care is multi-faceted and influenced by physical, mental, social, and environmental factors. Because males traditionally underutilize preventive healthcare services, self-care is increasingly important to manage disease symptomatology and slow disease progression. This study examines factors associated with barriers to disease self-care among Hispanic and non-Hispanic Black men ages ≥65 years with ≥1 chronic condition. Data were analyzed from a national sample of 470 Hispanic (43.2%) and non-Hispanic Black (56.8%) men and collected with an internet-delivered questionnaire. The 5-item Barriers to Self-Care Scale served as the dependent variable (range=5-20). A least squares regression model was fitted to identify factors associated with barriers to disease self-care. The model adjusted for sociodemographics, disease characteristics, health status, and social engagement and support. On average, participants were age 70.1 (±4.5) years and reported 3.9 (±2.6) chronic conditions. Men with higher body mass index (β=0.13, P=0.001), more depressive symptomatology (β=0.09, P=0.046), and lower self-rated quality of life (β=-0.23, P< 0.001) reported higher barriers to disease self-care. Further, men with poorer access to healthcare (β=-0.17, P< 0.001) and higher healthcare frustrations (β=0.17, P< 0.001) reported higher barriers to disease self-care. Barriers to self-care were positively associated with being more socially disconnected (β=0.09, P=0.049) and higher reliance on others to manage health problems (β=0.14, P< 0.001). Findings reinforce the importance of mental health, healthcare access, and social support for disease self-management among Hispanic and non-Hispanic Black men. Efforts are needed in clinical and community-based settings to reduce self-care barriers through culturally appropriate self-management education, interventions, and services.

